# Development and validation of case-finding algorithms for recurrence of breast cancer using routinely collected administrative data

**DOI:** 10.1186/s12885-019-5432-8

**Published:** 2019-03-08

**Authors:** Yuan Xu, Shiying Kong, Winson Y. Cheung, Antoine Bouchard-Fortier, Joseph C. Dort, Hude Quan, Elizabeth M. Buie, Geoff McKinnon, May Lynn Quan

**Affiliations:** 1 Department of Surgery, Foothills Medical Centre, University of Calgary, 3280 Hospital Drive NW, Calgary, Alberta T2N4Z6 Canada; 2 Department of Community Health Science, Foothills Medical Centre, University of Calgary, 3280 Hospital Drive NW, Calgary, Alberta T2N4Z6 Canada; 3 Department of Oncology, University of Calgary, Tom Baker Cancer Centre, 3280 Hospital Drive NW, Calgary, Alberta T2N4Z6 Canada; 4 The Ohlson Research Initiative, Arnie Charbonneau Cancer Institute, University of Calgary, 3280 Hospital Drive NW, Calgary, T2N4Z6 Alberta Canada

**Keywords:** Breast cancer recurrence, Validation study, Case-finding algorithm

## Abstract

**Background:**

Recurrence is not explicitly documented in cancer registry data that are widely used for research. Patterns of events after initial treatment such as oncology visits, re-operation, and receipt of subsequent chemotherapy or radiation may indicate recurrence. This study aimed to develop and validate algorithms for identifying breast cancer recurrence using routinely collected administrative data.

**Methods:**

The study cohort included all young (≤ 40 years) breast cancer patients (2007–2010), and all patients receiving neoadjuvant chemotherapy (2012–2014) in Alberta, Canada. Health events (including mastectomy, chemotherapy, radiation, biopsy and specialist visits) were obtained from provincial administrative data. The algorithms were developed using classification and regression tree (CART) models and validated against primary chart review.

**Results:**

Among 598 patients, 121 (20.2%) had recurrence after a median follow-up of 4 years. The high sensitivity algorithm achieved 94.2% (95% CI: 90.1–98.4%) sensitivity, 93.7% (91.5–95.9%) specificity, 79.2% (72.5–85.8%) positive predictive value (PPV), and 98.5% (97.3–99.6%) negative predictive value (NPV). The high PPV algorithm had 75.2% (67.5–82.9%) sensitivity, 98.3% (97.2–99.5%) specificity, 91.9% (86.6–97.3%) PPV, and 94% (91.9–96.1%) NPV. Combining high PPV and high sensitivity algorithms with additional (7.5%) chart review to resolve discordant cases resulted in 94.2% (90.1–98.4%) sensitivity, 98.3% (97.2–99.5%) specificity, 93.4% (89.1–97.8%) PPV, and 98.5% (97.4–99.6%) NPV.

**Conclusion:**

The proposed algorithms based on routinely collected administrative data achieved favorably high validity for identifying breast cancer recurrences in a universal healthcare system in Canada.

**Electronic supplementary material:**

The online version of this article (10.1186/s12885-019-5432-8) contains supplementary material, which is available to authorized users.

## Background

In the era of precision medicine, overall survival alone is not an adequate endpoint for assessing healthcare quality, comparing treatment efficacy, or informing decision making for patients with cancer, especially for cancers with long survival times such as breast cancer. Rather, examining recurrence free survival (RFS) is frequently important because it provides more relevant information regarding treatment- and cancer-specific outcomes. However, cancer recurrences are not explicitly documented in administrative data such as the cancer registry [1, 2], even though it represents a widely used data source for high volume, population-based, multi-institutional research. This poses considerable challenges for health researchers who need to effectively and reliably ascertain cancer recurrences to address their clinical question.

Cancer recurrences are events that usually require intensive health care resources. This may be reflected by an increase in medical encounters (i.e., oncologist or surgeon visits, hospitalizations, emergency room visits, etc.) or by the receipt of specific procedures (i.e., re-operation) and subsequent treatments (i.e., chemotherapy or radiation after the initial curative treatment). Therefore, routinely collected administrative data can be potentially used for identifying recurrences. Several studies have addressed this issue by using diagnosis and procedure codes to detect cases of cancer recurrence in selected populations of the U.S. health system [3–5]. However, these algorithms are not generalizable to different health systems because codes that form the algorithms are typically unique to the particular data coding system. In addition, in a private-market dominated health system, patterns of patient medical encounters may also be influenced by insurance status [6–8].

Therefore, this study aims to develop algorithms to detect breast cancer recurrences using routinely collected administrative data from Canada with a universal, single payer health system. Such an algorithm has the potential to be implemented in future data repositories to facilitate studies of disease surveillance, monitoring, and quality assessment.

## Method

### Study population and data sources

Data were derived from two prior population-based cohort studies of breast cancer patients with known high recurrence rates. The young women cohort consisted of all patients who were aged less than or equal to 40 years and diagnosed with breast cancer between 2007 and 2010 in Alberta, Canada [9]. The neoadjuvant chemotherapy cohort consisted of patients who were diagnosed with breast cancer between 2012 and 2014 and received neoadjuvant chemotherapy in the province [10]. Patients who did not have an Alberta healthcare number, emigrated from the province within 1 year of surgery, had more than one type of primary tumor, or had stage IV breast cancer were excluded.

Given the universal health system in Alberta, Canada, the provincial administrative data captured all patient medical encounters. The patient disease trajectory including hospitalizations, emergency department visits, clinic visits, surgeries, mammography or other breast imaging tests, biopsies, and endpoint status (i.e., deceased) after the primary treatment for breast cancer were obtained. The Alberta cancer registry (ACR) was used to ascertain the tumor characteristics (e.g., tumor stage, histology and molecular subtypes) and the dates of cancer diagnosis and primary treatment. The discharge abstract data (DAD), national ambulatory care reporting system (NACRS), and physician billing claims were extracted to determine the hospital admission, emergency department and clinic visits. The vital statistics data was linked to obtain the cause of death. The Alberta cancer measurement outcomes research and evaluation (C-MORE) data was linked to acquire the dates of the episodes of chemotherapy and radiation therapy.

In the two previous cohort studies, the recurrence status and recurrence date were ascertained by chart review and served as the reference against which to validate the proposed algorithms. Considering that the two prior studies only covered follow-up to 2015, we conducted additional chart review to ensure that follow-up was updated to September 1, 2017. A recurrence was defined as an in-situ or invasive tumor in the breast, lymph nodes, or at a distant site occurring 180 days or more after the primary treatment. This included patients with second primary breast cancer, but patients with more than one type of primary tumor (i.e., second primary non-breast tumors) were excluded from the cohort.

### Study variables

All clinically relevant variables were considered, however, we restricted variable selection to those that were commonly available in the administrative data to maximize generalizability of the algorithms. The specifications and classification codes for the variables are displayed in Additional file 1: Table S1.

#### A second round of chemotherapy

We assumed that breast cancer patients who underwent a second round of chemotherapy after primary treatment were more likely a recurrent case than those who did not. We counted the number of episodes of chemotherapy that occurred within a specific time frame after the primary treatment. An episode refers to a separate administration of chemotherapy. Considering that the recurrent breast cancer patient rarely receives a new round of chemotherapy within 6 months of primary treatment, we tested 180, 365 and 540 days after the primary treatment. More than two chemotherapy episodes within the pre-defined time frame was deemed as an indicator of recurrence.

#### Relevant diagnostic procedures or local treatment

Based on the assumption that patients who undergo a second diagnostic procedure or surgery or radiation treatment would have a higher risk of recurrence than those who did not, we built indicator variables for the relevant examinations (e.g., breast biopsy or mammography) or a second local treatment (e.g., mastectomy or radiation therapy) after the initial treatment. We also tested various time windows for these indicator variables including 180, 365 and 540 days after the primary treatment.

#### A second cluster of visits to oncologists

We further assumed that patients with frequent (or a change in the frequency of) visits to a cancer center after the completion of primary cancer treatment may also indicate a recurrence. Therefore, the second cluster of visits was flagged if the interval (days) between two visits was more than a pre-set value (180, 365 or 540 days). Next, the counts of visits within the determined cluster was inputted as an indicator in the algorithms.

#### Patient and tumor related potential indicators

Because breast cancer patients who are young and who have advanced-stage tumor tend to recur, we included age at diagnosis and tumor stage in the algorithms. In addition, surgery type and death caused by cancer were also included in the algorithm given that they are potentially indicative of recurrence.

### Statistical analyses

Descriptive statistics were used to characterize the study cohort. Continuous data were compared using T-test. Chi-squared or Fisher exact test was used for comparison of categorical variables. A randomly selected 60% set from the entire cohort served as training dataset; and the remain 40% of the study cohort used for validating the proposed algorithms. The classification and regression tree (CART) models [11, 12] were used for development of the algorithms considering that CART examines all potential binary classification thresholds and interactions among the indicator variables. In addition, CART provide us to freedom to set the penalty for misclassifications, thus, we can develop different algorithms to optimize one of the model performance metrics (e.g., sensitivity). All constructed indicator variables were inputted and tested for algorithm development. Each indicator variable divided the tree into two branches.

The splitting process stopped when fewer than 10 patients were left in the final node, or no more splits could be made (i.e., all classified patients were in the same group either recurrent or non-recurrent). CART modeling chose a splitting node (input variable) that minimized the Gini index which was most commonly used index for classification-type problems. The Gini index reached minimum value when the node made a perfect classification (i.e., all patients were correctly classified into recurrent or non-recurrent) and achieved maximum value when the node made a poor classification (i.e., none is correctly classified). The tree with the minimum sum of the Gini indices of each node was the best-performing tree. However, to avoid overfitting our data and to simplify the algorithm, we chose the tree that contained the fewest nodes and maintained a misclassification rate that fell within one standard error of the misclassification rate of the best-performing tree [11].

We then validated the algorithms against the chart review data by measuring sensitivity, specificity, positive predictive value (PPV), negative predictive value (NPV), accuracy. The 95% confidence intervals of the validity measures were calculated assuming an exact binomial distribution.

To meet different research needs [13–17] we developed a set of algorithms which prioritized sensitivity, PPV or accuracy, respectively. To achieve this, we assessed the CART-generated algorithms by excluding the node (or variable) that contributed marginally to the targeted metric (e.g., “age” was excluded from the tree because it provided very limited classification contribution and made the tree complex) and adjusting the cost for misclassification to optimize the target accuracy measurement (such as PPV) while maintaining a reasonable threshold for the other measurement domains (i.e., sensitivity, specificity and NPV). We also built an algorithm with balanced sensitivity and PPV, which means both sensitivity and PPV reached the highest levels concurrently in one algorithm. Similarly, we developed an algorithm with balanced specificity and NPV. Furthermore, we developed and validated the method by combining the algorithm with high sensitivity and the algorithm with high PPV (plus additional chart review for discordant cases) to identify recurrences.

Data preparation and descriptive analysis were conducted using SAS 9.4 (SAS Institute Inc., Cary, NC), and CART model development was performed using CART (Salford Systems, San Diego, CA).

## Results

In total, 598 patients with stage 0 to III breast cancer were included in analyses. This cohort consisted of 282 (47.2%) young patients (≤ 40 years old) and 316 (52.8%) neoadjuvant chemotherapy patients (Table 1). Among the 598 patients, we observed 20.2% (121) recurrences during a median follow-up of 4 (Interquartile range 3–5) years. Tumor stage, grade, HER2 (Human Epidermal growth factor Receptor 2) status, primary surgery type and cancer specific death and overall death were significantly different between recurrent and non-recurrent patients, whereas tumor characteristic such as histology subtype and patient/treatment characteristics including age, adjuvant therapy, follow-up length between the recurrent and non-recurrent patients were not significantly different (Table 1).

**Table 1:** description of the patient characteristics, treatments and other variables

Characteristic	All women (*N* = 598)	Women who did not have recurrence (*N* = 477)	Women who had recurrence (*N* = 121)	*P* value
Age, Median (IQR), year	40 (36–53)	40 (36–54)	40 (36–50)	0.272
Study follow-up, Median (IQR), year	4 (3–5)	4 (2–5)	4 (3–5)	0.091
Year of Diagnosis				0.33
2007–2009	259 (43.3)	208 (43.6)	51 (42.1)	
2010–2012	192 (32.1)	147 (30.8)	45 (37.2)	
2013–2015	147 (24.6)	122 (25.6)	25 (20.8)	
Stage				<.0001
0	2 (0.3)	2 (0.4)	0 (0)	
I	93 (15.6)	84 (17.6)	9 (7.4)	
II	311 (52.0)	270 (56.6)	41 (33.9)	
III	192 (32.1)	121 (25.4)	71 (58.7)	
Tumor grade				0.012
1	32 (5.3)	32 (6.7)	0 (0.0)	
2	211 (35.3)	167 (35.0)	44 (36.4)	
3	342 (57.2)	270 (56.6)	72 (59.5)	
Unknown	13 (2.2)	8 (1.7)	5 (4.1)	
Histology				0.352
Ductal	538 (90.0)	432 (90.6)	106 (87.6)	
Lobular	27 (4.5)	22 (4.6)	5 (4.1)	
Ductal lobular mixed	10 (1.7)	6 (1.3)	4 (3.3)	
Other	23 (3.8)	17 (3.5)	6 (5.0)	
ER status				0.266
Positive	439 (73.4)	355 (74.4)	84 (69.4)	
Negative	159 (26.6)	123 (25.6)	37 (30.6)	
PR status				0.216
Positive	375 (62.7)	305 (63.9)	70 (57.8)	
Negative	223 (37.3)	172 (36.1)	51 (42.2)	
HER2 status				0.028
Positive	161 (26.9)	138 (28.9)	23 (19.0)	
Negative	437 (73.1)	339 (71.1)	98 (81.0)	
Surgery				0.003
No surgery	5 (0.8)	4 (0.8)	1 (0.8)	
BCS	159 (26.6)	141 (29.6)	18 (14.9)	
Mastectomy	434 (72.6)	332 (69.6)	102 (84.3)	
Chemotherapy				0.614
Yes	547 (91.5)	436 (91.4)	111 (91.8)	
No	40 (6.7)	31 (6.5)	9 (7.4)	
Unknown	11 (1.8)	10 (2.1)	1 (0.8)	
Radiation therapy				0.587
Yes	194 (32.4)	150 (31.5)	44 (36.4)	
No	209 (35.0)	169 (35.4)	40 (33.0)	
Unknown	195 (32.6)	158 (33.1)	37 (30.6)	
Hormone therapy				0.396
Yes	214 (35.8)	177 (37.1)	37 (30.6)	
No	192 (32.1)	149 (31.2)	43 (35.5)	
Unknown	192 (32.1)	151 (31.7)	41 (33.9)	
Death				<.0001
Yes	92 (15.4)	13 (2.7)	77 (63.6)	
No	506 (84.6)	464 (97.3)	44 (36.4)	
Cause of death				<.0001
Death due to breast cancer	76 (12.7)	10 (2.1)	66 (54.5)	
Death due to other causes	16 (2.7)	3 (0.6)	11 (9.1)	
Alive	506 (84.6)	464 (97.3)	44 (36.4)	

*IQR* interquartile range, *ER* estrogen receptor, *PR* progesterone receptor, *HER2* Human Epidermal growth factor Receptor 2

**Fig. 1:**
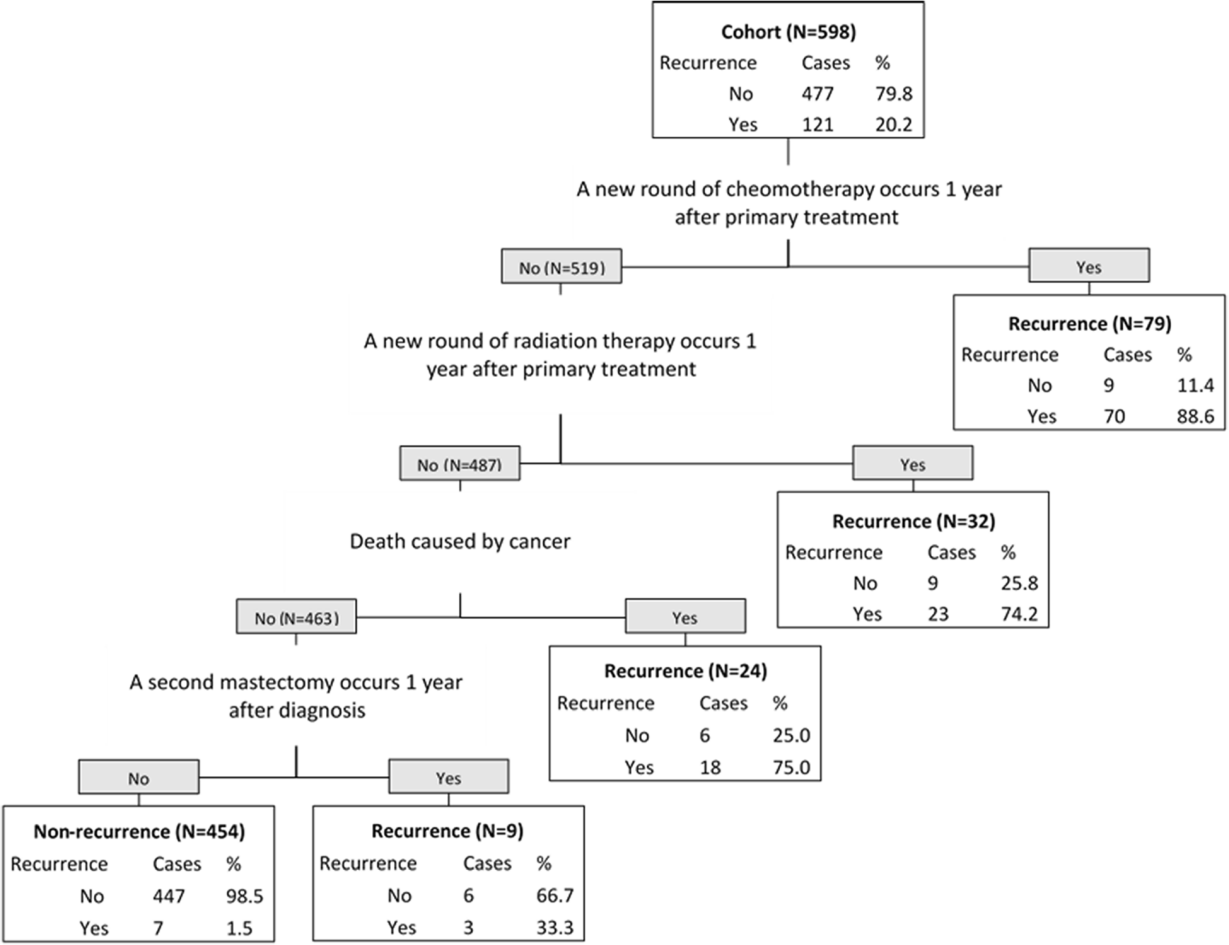
the algorithm with high sensitivity for identifying recurrence of breast cancer. “Yes” means the criteria was met; “No” means the criteria was not met

**Fig. 2:**
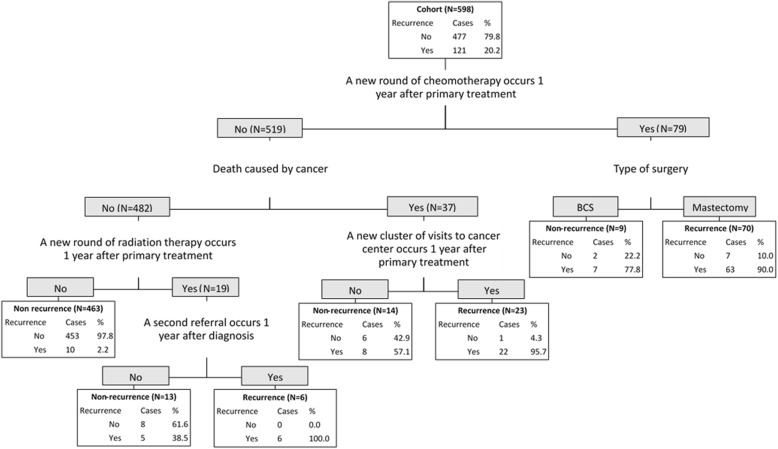
the algorithm with high positive predictive value for identifying recurrence of breast cancer. “Yes” means the criteria was met; “No” means the criteria was not met

**Fig. 3:**
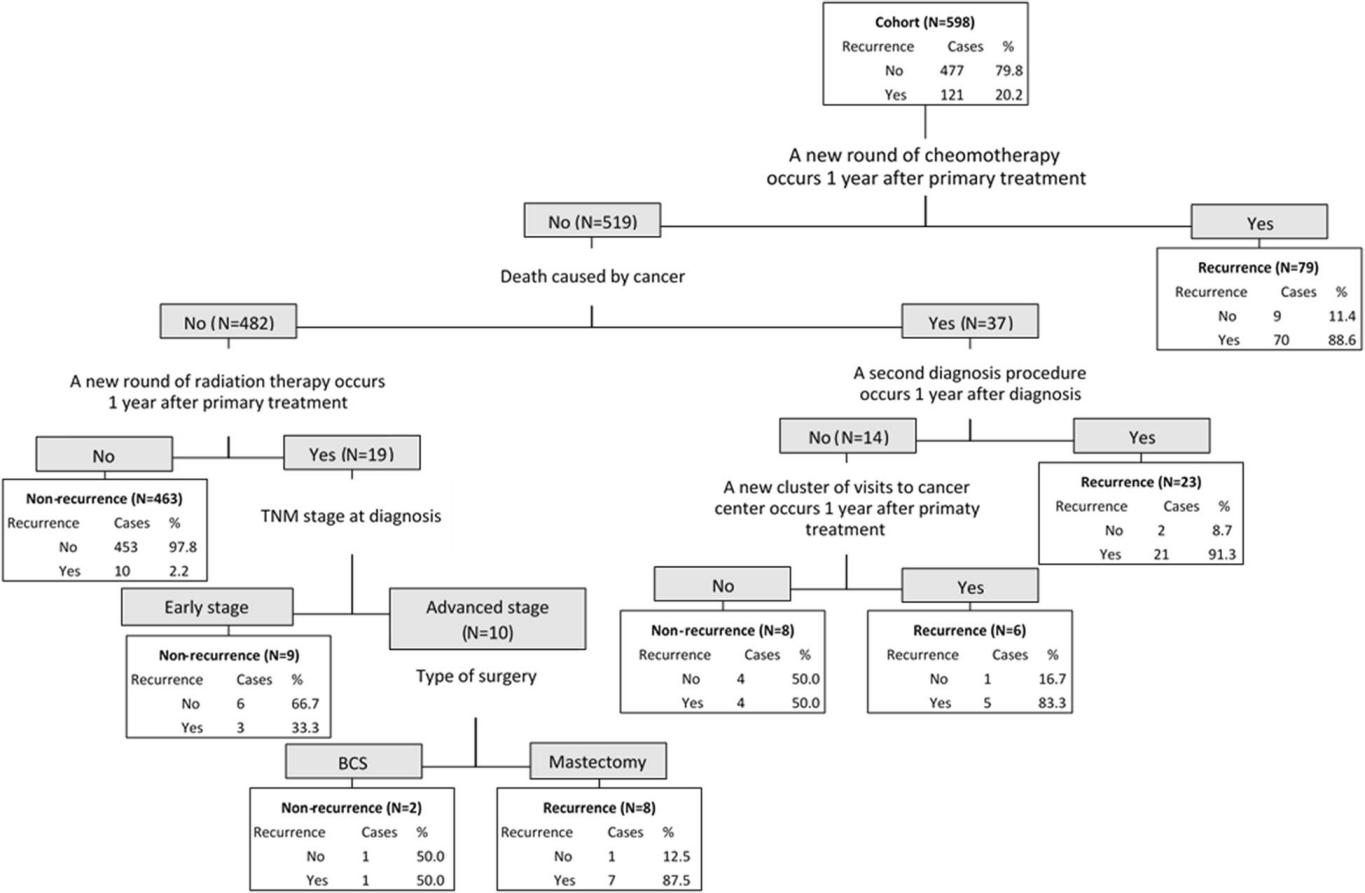
the algorithm with high overall accuracy for identifying recurrence of breast cancer. “Yes” means the criteria was met; “No” means the criteria was not met

The performance of developed algorithms is presented in Table 2 and the detailed classification trees are depicted in Figs. 1, 2, 3 and Additional file 1. Since the validation results were similar in testing set and in the entire cohort, we presented the performance results of the algorithms in the entire cohort and attached the validation results in the testing set in the Additional file 1: Table S2. The high sensitivity algorithm achieved 94.2% (95% CI: 90.1–98.4%) sensitivity, 93.7% (91.5–95.9%) specificity, 79.2% (72.5–85.8%) PPV, 98.5% (97.3–99.6%) NPV, and 93.8% (91.9–95.7%) accuracy. The high PPV algorithm obtained 75.2% (67.5–82.9%) sensitivity, 98.3% (97.2–99.5%) specificity, 91.9% (86.6–97.3%) PPV, 94% (91.9–96.1%) NPV, and 93.6% (91.7–95.6%) accuracy. The high accuracy algorithm reached 85.1% (78.8–91.5%) sensitivity, 97.3% (95.8–98.7%) specificity, 88.8% (83.1–94.5%) PPV, 96.3% (94.6–98.0%) NPV, and 94.8% (93.0–96.6%) accuracy.

**Table 2:** the validity of the algorithms

Algorithm	Sensitivity	Specificity	PPV	NPV	Accuracy
	(%, 95 CI)	(%, 95 CI)	(%, 95 CI)	(%, 95 CI)	(%, 95 CI)
High sensitivity	94.2	93.7	79.2	98.5	93.8
	(90.1–98.4)	(91.5–95.9)	(72.5–85.8)	(97.3–99.6)	(91.9–95.7)
High PPV	75.2	98.3	91.9	94	93.6
	(67.5–82.9)	(97.2–99.5)	(86.6–97.3)	(91.9–96.1)	(91.7–95.6)
High accuracy	85.1	97.3	88.8	96.3	94.8
	(78.8–91.5)	(95.8–98.7)	(83.1–94,5)	(94.6–98)	(93–96.6)
Balanced sensitivity and PPV	89.3	96.2	85.7	97.2	94.8
	(83.7–94.8)	(94.5–97.9)	(79.6–91.8)	(95.8–98.7)	(93.0–96.6)
Balanced specificity and NPV	91.7	94.5	81.0	97.8	94.0
	(86.8–96.6)	(92.5–96.6)	(74.5–87.6)	(96.5–99.2)	(92.1–95.9)
Combining high sensitivity and high PPV algorithms plus chart review	94.2	98.3	93.4	98.5	97.5
	(90.1–98.4)	(97.2–99.5)	(89.1–97.8)	(97.4–99.6)	(96.2–98.7)

*CI* confidence interval, *PPV* positive predictive value, *NPV* negative predictive value

For the algorithm with high sensitivity (Fig. 1), the indicator variable for “a new round of chemotherapy” divided the whole cohort (598) into two groups. This meant that women (*N* = 79) who underwent a new round of chemotherapy at 1 year or more after the primary treatment (we tested three timeframes180 days, 365 days or 540 days and found that the 365-day resulted in the highest performance) were classified as a recurrent case. Among the 79 women, 89% actually experienced a recurrence. In the women (*N* = 519) who did not meet the above criteria, they were regarded as recurrent if they underwent a new round of radiation therapy at 1 year or more after the primary treatment. Using this criterion, 32 women met this recurrence definition and 487 did not. Among the 32 women, 23 (74.2%) were correctly identified. For the remaining 487 women, 24 were classified as recurrent because they had died due to the cancer. Of the 24 cases, 18 (75%) were correctly identified. Finally, among the 463 women who did not die from cancer, 9 patients were classified as recurrent because they had a mastectomy after the primary treatment. All other classification figures can be interpreted following the above interpretation of Fig. 1.

The algorithm with balanced sensitivity and PPV achieved 89.3% (83.7–94.8%) sensitivity, 96.2% (94.5–97.9%) specificity, 85.7% (79.6–91.8%) PPV, 97.2% (95.8–98.7%) NPV, and 94.8% (93.0–96.6%) accuracy (Table 2 and Additional file 1: Figure S1). The algorithm with balanced specificity and NPV had 91.7% (86.8–96.6%) sensitivity 94.5% (92.5–96.6%) specificity, 81.0% (74.5–87.6%) PPV, 97.8% (96.5–99.2%) NPV, and 94.0% (92.1–95.9%) accuracy (Table 2 and Additional file 1: Figure S2).

The method of combining the high sensitivity and high PPV algorithms resulted in 7.5% (45 out of 598) discordant cases. If additional chart review was conducted to resolve the discordances, the accuracy metrics would be improved to 94.2% (90.1–98.4%) sensitivity, 98.3% (97.2–99.5%) specificity, 93.4% (89.1–97.8%) PPV, 98.5% (97.4–99.6%) NPV and 97.5% (96.2–98.7%) accuracy (Table 2).

## Discussion

To our knowledge, this represents the first study to develop algorithms for identifying breast cancer recurrences using administrative data from a publicly funded health system. Currently, chart review is the only reliable way to obtain recurrence status, but this approach is time-consuming and inefficient. The proposed algorithms have the potential to greatly reduce the resources needed to identify recurrences in a large population. The algorithms may impact future health services research, including the facilitation of quality improvement or effectiveness studies in addition to population health studies. Moreover, this study also provides a potential framework for constructing similar algorithms to identify recurrences for other cancers using administrative data from a health system with universal insurance coverage.

Earle et al. developed algorithms for identifying relapse of acute myelogenous leukemia based on diagnosis and procedure codes from SEER (Surveillance, Epidemiology, and End Results) and Medicare data [18]. The algorithms were validated in an 89-patient cohort and the best algorithm achieved 86% sensitivity, 99% specificity, 95% PPV and 96% NPV. However, this prior study was not population-based, and the coding systems were different from those in Canada, which used the Current Procedural Terminology (CPT) and Health Care Common Procedure Coding System (HCPCS). Chubak et al. [3] developed algorithms by using administrative data (including diagnosis codes, procedure codes, prescriptions, and cancer registry data) of a selected population that was enrolled in Group Health in Washington, U.S., and reported an algorithm with high sensitivity and high PPV which had a similar accuracy to ours. However, these previous algorithms mainly relied on specific cancer registry records or cancer diagnosis codes that highlight the existence of secondary malignant tumors. This information is limited in Canadian administrative data. Due to the lack of universal health care coverage in the U.S., we can also assume that the patterns of care that may signify a recurrence in the U.S. may not be completely reflected in non-U.S. data. Importantly, the algorithms that were developed in this present study are based on common data elements that exist in most sources of administrative data, which ensure potential broad application of these algorithms in Canada and other jurisdictions with universal healthcare systems.

We developed several different algorithms prioritizing different performance measures to meet various research needs [15, 19]. The high sensitivity algorithm is preferred for studies where the aim is to identify all patients with a given condition. For example, for one study that is focused on the surveillance of the adverse events of a specific treatment for breast cancer recurrence in a population, the high sensitivity algorithm would be ideal to capture as many recurrence cases as possible as a first step; then further determining method can be applied to figure out the incidence of the adverse events. The high PPV algorithm is important when the purpose of a study is to ensure that all identified cases are truly positive. For example, if one wants to accurately recruit a cohort of patients with breast cancer recurrence to investigate the patterns of care and survivorship. The balanced sensitivity and PPV algorithm is suitable for the study that desires to capture as many recurrence cases as possible and also ensure a high proportion of real recurrence among the captured cases, such as the study of incidence or prevalence of breast cancer recurrence in a given population. Conversely, the balanced specificity and NPV algorithm fits the study that aims to investigate the effect of a prevention treatment for a cohort of non-recurrent patients. The high accuracy algorithm is supposed to fit all above scenarios (if no better algorithm), but we recommended the method of combining high sensitivity and high PPV algorithms plus chart review (to resolve the discordant results from the two algorithms) given that it overperformed the high accuracy and all other algorithms.

There are several points worthy of attention regarding the utilization and generalizability of the algorithms. First, given that the time intervals associated with recurrence vary, we tested several time intervals for the time-dependent variables indicative of recurrence in the natural clinical course, such as second round of chemotherapy or radiation after primary treatment. Clinically, the primary treatment duration for a patient undergoing surgery, chemotherapy and radiation is completed in no less than 6 months, time intervals beyond 180 days were selected for testing in the algorithms. Among the tested intervals we found that 365-day worked best. It is possible that if applied to other cohorts, more granular intervals (e.g., 300, 320, or 380 days) maybe work better than 365 days however, this would require validation for a particular cohort. Thus, we recommend using the interval of 365 days if the users do not plan to do validation. Interested users performing a validation can tailor the proposed algorithm by testing various intervals and choosing the optimal one. Second, the contributions of the included indicator variables in our algorithms varied. For example, ‘cause of death’ contributed more than ‘age’ in our algorithms; this may pose concern on the generalizability of the algorithms. When applied to other populations with different disease characteristics, while the classification power of each node may change, the performance of the overall model may be not compromised, because each of the algorithms is a “decision tree” consisting of several criteria which work together to identify recurrence, instead of relying on one single criterion. Third, although external validation is needed before wide application of the algorithms, we believe that our algorithm is potentially applicable to other Canadian data based on following reasons: 1) the universal health system and similar patterns of care; 2) the similarities in administrative data between provinces in Canada; 3) to avoid overfitting, we followed the standard algorithm development practice of splitting the cohort into training and testing sets. 4) we only included “generic” data elements that commonly exist in other administrative datasets in the models; 5) our study cohort included patients with varying tumor characteristics (including ER-positive patients, and patients only received hormone therapy).

Several limitations should be considered when applying the algorithms. First, the study cohort was determined by previously collected chart review data of two population-based cohorts with high risk of breast cancer recurrence, so the application of the proposed algorithms in the general breast cancer population requires validation. However, the cornerstone of the proposed algorithm is the pattern of medical encounters of patients with recurrent breast cancer, so we hypothesize that this might be generalizable to most breast cancer patients. Second, the study only used data from one province with relatively small sample size, so external validation is needed by testing the algorithms in other jurisdictions with universal health coverage. Finally, our algorithms were not designed to distinguish second primary breast cancers from recurrences. However, both of these endpoints are usually considered together in many breast cancer studies because both are equally important in the assessment of outcomes in breast cancer [20–22].

## Conclusion

The proposed algorithms achieved favourably high validity for identifying breast cancer recurrences using widely available administrative data in a universal health system in Canada. Further work is needed for external validation of the algorithms prior to their widespread use.

## Additional file

Additional file 1: **Table S1.** The specifications and codes for the indicator variables. **Table S2.** The validity results of the algorithms in the testing set (40% of the entire cohort). **Figure S1.** the algorithm with balanced sensitivity and positive predictive value for identifying recurrence of breast cancer. “Yes” means the criteria was met; “No” means the criteria was not met. **Figure S2.** the algorithm with balanced specificity and negative predictive value for identifying recurrence of breast cancer. “Yes” means the criteria was met; “No” means the criteria was not met. (DOCX 710 kb)

## Abbreviations

ACR: Alberta cancer registry
CART: Classification and regression tree
C-MORE: Cancer measurement outcomes research and evaluation
CPT: Current Procedural Terminology
DAD: Discharge abstract data
HCPCS: Health Care Common Procedure Coding System
HER2: Human Epidermal growth factor Receptor 2
NACRS: National ambulatory care reporting system
NPV: Negative predictive value
PPV: Positive predictive value
RFS: Recurrence free survival
SEER: Surveillance, Epidemiology, and End Results
