# Supplementary material for: Development and validation of case-finding algorithms for recurrence of breast cancer using routinely collected administrative data
Source: BMC Cancer. 2019 Mar 8;19:210. doi: 10.1186/s12885-019-5432-8 (PMC6408837; doi:10.1186/s12885-019-5432-8)
Supplement: Supplementary file 1 — Table S1. The specifications and codes for the indicator variables. Table S2. The validity results of the algorithms in the testing set (40% of the entire cohort). Figure S1. the algorithm with balanced sensitivity and positive predictive value for identifying recurrence of breast cancer. “Yes” means the criteria was met; “No” means the criteria was not met. Figure S2. the algorithm with balanced specificity and negative predictive value for identifying recurrence of breast cancer. “Yes” means the criteria was met; “No” means the criteria was not met. (DOCX 710 kb) [file 12885_2019_5432_MOESM1_ESM.docx]

Supplementary Table 1 study variables specifications and classification codes

| **Variables** | **Specifications/Codes** |
| --- | --- |
| Breast surgery/procedure |  |
| Mastectomy | CCI: 1YM89^^, 1YM90^^, 1YM91^^,1YM92^^  Alberta physician billing codes: 97.12A, 97.12B, 97.21A, 97.22A |
| Breast conservative surgery | CCI: 1YM87^^, 1YM88^^  Alberta physician billing codes: 97.27A, 97.27B |
| Breast biopsy | CCI: 2JZ71^^, 2YM71^^, 2MD71^^, 2YR71^^, 2YS71^^  Alberta physician billing codes: 97.81, 97.82A, 97.89A, 97.11A, 97.11B |
| Mammography | CCI: 3YM10^^  Alberta physician billing codes: X26 |
| Chemotherapy | Using the date of each administration of chemotherapy |
| Radiation therapy | Using the date of each radiation therapy |
| Referral to oncologist | Using the date of each referral to oncologist |
| Type of medical visit |  |
| Cancer center visit | Based on the service facility we identified the cancer center visit. |
| Specialty visits | Based on the provider classification we identified the type of specialty including the oncologists and general surgeons. |
| Primary cancer site (breast cancers) | ICD-O: C50.^ and D05.^  ICD-9: 174.^ and 233.^  ICD-10: C50.^ and D05.^ |
| Death caused by cancer | ICD-9: 140.^ - 208.^ and 233^  ICD-10: C00.^ - C97.^ and D05.^ |

CCI: Canadian Classification of Health Intervention; ICD-9: International Classification of Disease – ninth edition; ICD-10: International Classification of Disease – tenth edition; ICD-O: International Classification of Disease for Oncology;

Note: Physician billing codes are derived from the Alberta Schedule of Medical Benefits (SOMB).

Supplementary Table 2 The validity results of the algorithms in the testing set (40% of the entire cohort)

|  | **Sensitivity** | **Specificity** | **PPV** | **NPV** | **Accuracy** |
| --- | --- | --- | --- | --- | --- |
| **High sensitivity** | 94.1 (87.7-100) | 92.8 (89.2-96.4) | 77.4 (67-87.8) | 98.4 (96.5-100) | 93.1 (89.9-96.3) |
| **High PPV** | 74.5 (62.5-86.5) | 97.4 (95.2-99.7) | 89.1 (79.2-98.3) | 93.6 (90.2-97) | 92.7 (89.4-95.9) |
| **High accuracy** | 80.4 (69.5-91.3) | 96.9 (94.5-99.3) | 87.2 (77.7-96.8) | 95 (91.9-98) | 93.5 (90.4-96.6) |
| **Balanced sensitivity and PPV** | 90.2 (82-98.4) | 95.4 (92.4-98.3) | 83.6 (73.9-93.4) | 97.4 (95.1-99.6) | 94.3 (91.4-97.2) |
| **Balanced specificity and NPV** | 92.2 (84.8-99.5) | 92.8 (89.2-96.4) | 77 (66.5-87.6) | 97.8 (95.7-99.9) | 92.7 (89.4-95.9) |


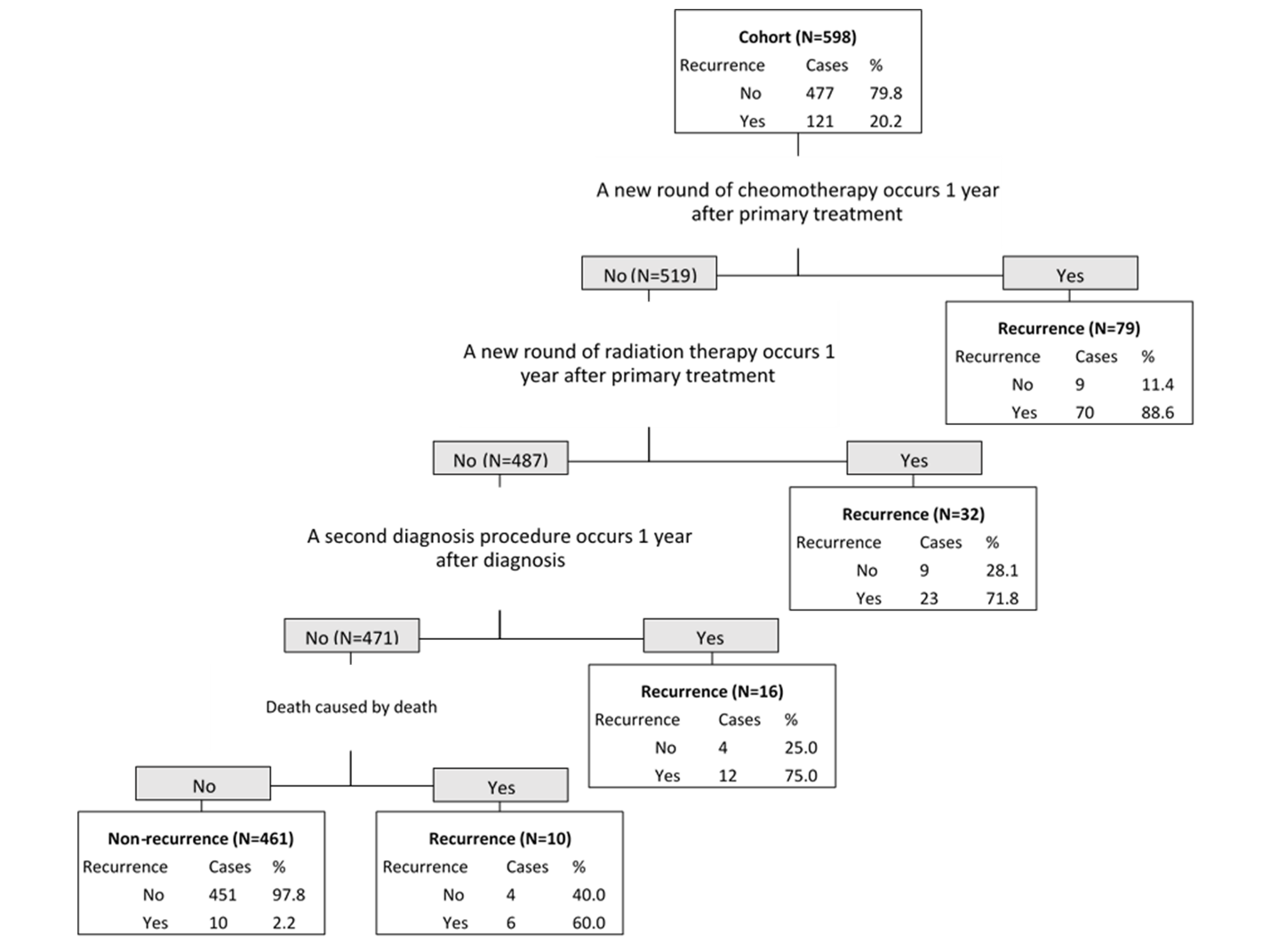


Supplementary figure 1 the algorithm with balanced sensitivity and positive predictive value for identifying recurrence of breast cancer. “Yes” means the criteria was met; “No” means the criteria was not met.


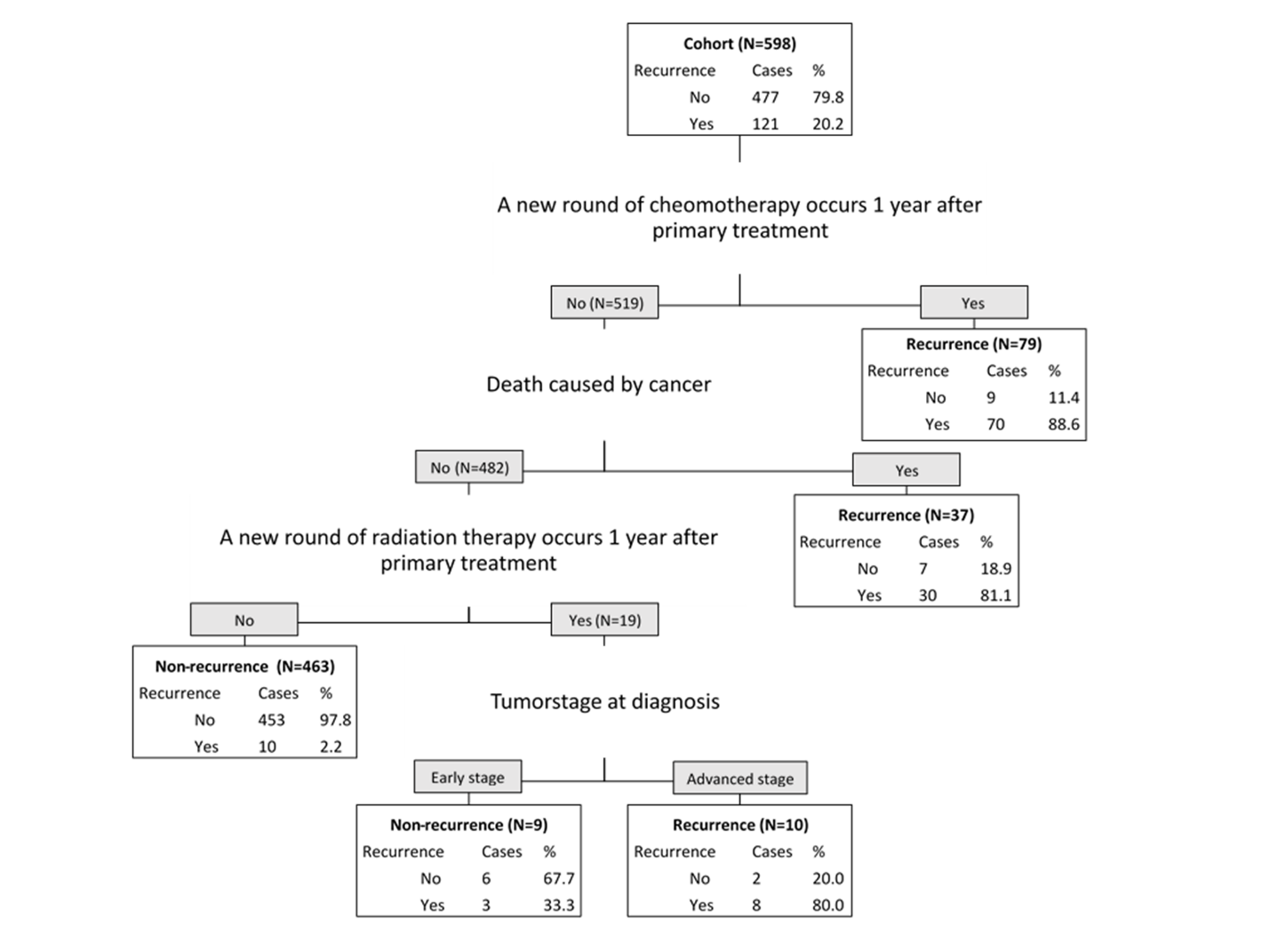


Supplementary figure 2 the algorithm with balanced specificity and negative predictive value for identifying recurrence of breast cancer. “Yes” means the criteria was met; “No” means the criteria was not met.
